# De Novo and Relapsing Glomerulonephritis following SARS-CoV-2 mRNA Vaccination in Microscopic Polyangiitis

**DOI:** 10.1155/2021/8400842

**Published:** 2021-10-22

**Authors:** Tamara Davidovic, Judith Schimpf, Hannelore Sprenger-Mähr, Armin Abbassi-Nik, Afschin Soleiman, Emanuel Zitt, Karl Lhotta

**Affiliations:** ^1^Department of Internal Medicine III (Nephrology and Dialysis), Feldkirch Academic Teaching Hospital, Feldkirch, Austria; ^2^Pathology, Cytodiagnostics and Molecular Pathology, Hall in Tirol, Austria

## Abstract

Vaccination against SARS-CoV-2 is the most important advance in the fight against the ongoing coronavirus pandemic. Recent case reports show that the SARS-CoV-2 vaccines can very rarely cause de novo or relapsing glomerular disease. Here, we report two female patients with microscopic polyangiitis, who developed severe glomerulonephritis after immunisation with the BNT162b2 mRNA vaccine. One patient with a possible ongoing but undiagnosed disease developed severe necrotising glomerulonephritis after the second vaccination. In the other patient with a long-lasting disease, rituximab maintenance therapy had been postponed because of the coronavirus pandemic. She noted macrohematuria immediately after the second vaccine dose and developed a severe renal relapse leading to end-stage kidney disease. We suggest that patients with ANCA-associated vasculitis be carefully monitored for disease activity immediately before and after receiving the SARS-CoV-2 vaccination, especially if maintenance therapy has been interrupted. Ultimately, mRNA vaccines should probably be avoided in these patients.

## 1. Introduction

The SARS-CoV-2 pandemic has important consequences for the outcome and management of patients suffering from kidney disease, not only those on dialysis or patients who received a transplant [1]. Likewise, patients suffering from glomerulonephritis and in need of immunosuppressive therapy may be prone to develop COVID-19. Thus, the clinician's dilemma is, on the one hand, to control the kidney disease and, on the other hand, to protect the patient from developing severe COVID-19 [2].The rapid development and distribution of SARS-CoV-2 vaccines was seen as a very welcomed development to solve this dilemma [3]. Patients on immunosuppressive treatment such as rituximab, however, may have an impaired or even absent antibody response upon vaccination [4]. Recently, another level of concern and complexity has been introduced by reports that these vaccines may induce or exacerbate glomerular diseases, mostly minimal change disease and IgA nephropathy (reviewed in [5]). To date, four cases of de novo ANCA-associated vasculitis after SARS-CoV-2 vaccination have been published: two with PR3-ANCA vasculitis following the second inoculation with the mRNA-1273 (Moderna) vaccine [6, 7]; one with MPO-ANCA renal-limited vasculitis after receiving the second dose of the BNT162b2 mRNA (Pfizer-BioNTech) vaccine [8]; and one with MPO-ANCA vasculitis after the first dose of the AZD1222 (Oxford-AstraZeneca) vaccine [9]. Here, we report two additional vasculitis patients with de novo and relapsing MPO-ANCA-associated glomerulonephritis after receiving the BNT162b2 mRNA (Pfizer-BioNTech) SARS-CoV-2 vaccine.

## 2. Case Presentations

### 2.1. Patient 1

**Figure 1:**
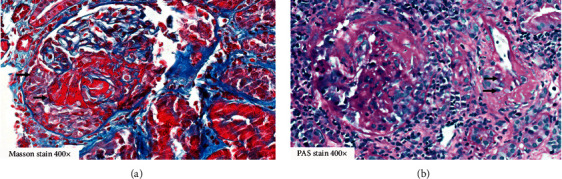
(a) Masson trichrome stain 400*x*; kidney core biopsy of patient 1 with glomerular capillary necrosis and acute extravasation of fibrin into Bowman's space with a fibrocellular crescent (arrows). (b) PAS stain of a glomerulus with rupture of Bowman's capsule and segmental tuft sclerosis. Adjacent segmental vessel wall necrosis of an interstitial arteriole (arrows) depicting highly acute microangiopathic vasculitis is shown.

A 54-year-old Caucasian female had suffered from diffuse arthralgia two years earlier. A diagnosis of seronegative polyarthritis was made. She quickly responded to a short course of oral steroids. Three months later, arthralgias recurred and a second prolonged course of steroids was necessary. Nine months ago, she complained of acute hearing loss, and right-sided otitis media requiring paracentesis was diagnosed. Two months ago, she received the first dose of the BNT162b2 mRNA vaccine. Five weeks later, she developed fatigue and her body temperature rose to 37.8°C. Her left eye was reddened and painful. A urinary analysis was not performed. Six weeks after the first, she received the second dose of the BNT162b2 mRNA vaccine. During the next two weeks, she felt very weak and complained of dizziness and loss of appetite. The patient was admitted to the hospital. Initial results of blood and urine chemistry are shown in Table 1. A test for MPO-ANCA was positive. A diagnosis of microscopic polyangiitis with renal involvement was made, and a kidney biopsy was performed. Histology revealed acute and chronic pauci-immune necrotising glomerulonephritis with tuft necroses and fibrocellular crescents in seven out of 21 glomeruli (Figure 1). A CT scan revealed discrete opacities in both the lungs. Her upper respiratory tract and eyes were normal. The patient was treated with oral methylprednisolone 60 mg per day, which led to immediate improvement of general symptoms, and rituximab, two doses of 1g two weeks apart. Six weeks later, her serum creatinine had improved from 187 *μ*mol/l to 151 *μ*mol/l.

### 2.2. Patient 2

A 78-year-old Caucasian woman had been diagnosed with microscopic polyangiitis nine years earlier. She had suffered from severe pulmonary haemorrhage. A renal biopsy revealed advanced acute and chronic crescentic necrotising glomerulonephritis with moderate interstitial fibrosis and tubular atrophy. She was treated with plasma exchange, iv, and oral methylprednisolone and six cycles of iv cyclophosphamide. Rituximab 500 mg was administered every six months for two and a half years. After another two and a half years without immunosuppression, an increase in MPO-ANCA titer, deterioration of renal function with nephritic sediment, and proteinuria were noted. A second renal biopsy established a diagnosis of advanced, but still active glomerulonephritis. She received induction therapy with 1g rituximab and oral methylprednisolone, and maintenance therapy with rituximab was recommenced. Renal function remained stable with an eGFR around 25 ml/min/1.73 m^2^, C-reactive protein was always negative, and the MPO-ANCA titer was declining. Consequently, it was decided to withhold further rituximab maintenance until vaccination or waning of the pandemic. The last dose had been administered nine months ago. One month before vaccination, MPO-ANCA increased from 16 IU/ml to 99 IU/ml. Her B-cell count was 3 cells/*μ*l. There were no clinical and laboratory signs of disease activity. The patient received two doses of the BNT162b2 mRNA SARS-CoV-2 vaccine three weeks apart. Two days after the second dose, she noted dark urine, arthralgia, fatigue, and nausea, which she considered a normal reaction to vaccination. Two months after the second dose, she was seen at our institution. Her serum creatinine had risen to 728 *μ*mol/L (Table 1). No other organ involvement was observed. B cells had risen to 21 cells/*μ*l and MPO-ANCA to >134 IU/ml. She was started on hemodialysis and received a short course of iv and oral methylprednisolone and 1g rituximab. Nevertheless, the patient remained dialysis dependent.

A detailed summary of all reported cases including ours is given in Table 2.

## 3. Discussion

These two case reports increased the number of patients suffering from new or relapsing ANCA vasculitis following SARS-CoV-2 vaccination to six. Our second patient is the first one with a disease relapse after vaccination. We suggest that both our patients were vaccinated in a vulnerable phase of their disease. Patient 1 had signs and symptoms of vasculitis for two years. The histological findings also suggest that she had already had renal flares. The strong immune response induced by vaccination as evidenced by a very high anti-SARS-CoV-2 spike protein antibody titer may have induced the severe exacerbation of renal disease.

The second patient illustrates the dilemma of disease control with immunosuppression, avoiding severe SARS-CoV-2 infection and vaccination. Being elderly, having chronic kidney disease stage G4A3, and being on rituximab maintenance, she was at very high risk for an adverse outcome if she contracted COVID-19. Rituximab has clearly been established as a major risk factor for COVID-19-related mortality in rheumatoid arthritis patients [10, 11]. At the time when her maintenance therapy was reappraised, Austria was hit by a second wave of the coronavirus pandemic with incidence rates up to 1,000 cases per 100,000 persons per week. She was clinically stable without symptoms of vasculitis. Therefore, we decided to withhold the next rituximab dose until the pandemic was brought under control or she was vaccinated [2]. Interrupting immunosuppression in ANCA vasculitis is not without risk. According to a recent report, rituximab was postponed in 21 out of 77 patients during the pandemic, and two of those showed evidence of a relapse. The risk of relapse far outweighed the risk of contracting COVID-19 [12]. Our patient experienced an increase in the MPO-ANCA titer and had signs of B-cell recovery one month before vaccination, both indicators of an increased risk of relapse. Vaccination was most likely the final trigger for disease exacerbation. Nevertheless, due to B-cell recovery, she had a moderate SARS-CoV-2 antibody response to immunisation.

Should patients treated with rituximab be immunized against SARS-CoV-2 at all? These vaccines, in addition to a strong antibody response, also induce significant cellular immunity against the virus [13, 14]. Cellular immunity has been demonstrated in rituximab-treated patients following vaccination [15]. Therefore, vaccines can be expected to offer at least some protection against severe disease, even in the absence of an antibody response. Timing of rituximab administration and vaccination is certainly important. It is suggested that vaccination be performed not earlier than six months after rituximab and that rituximab be given again as soon as four weeks after immunisation [16]. Clearly, B-cell recovery increases the likelihood of an antibody response [17]. But, it also puts vasculitis patients at risk of relapse, as exemplified by patient 2.

Whether influenza vaccination is associated with de novo or relapsing ANCA vasculitis was a matter of debate several years ago [18, 19]. Jeffs et al. reported a patient who developed PR3-ANCA vasculitis following influenza immunisation [20]. They found that ANCA production by peripheral blood mononuclear cells of the patient was enhanced on incubation with influenza vaccines contaminated with viral RNA but not with uncontaminated vaccines. They also showed that this effect was dependent on stimulation of toll-like receptor-7 (TLR7) by RNA. TLR7 is constitutively expressed in endosomes of dendritic cells and B cells and on activation by viral RNA induces a strong antiviral type 1 interferon response. Activation of TLR7 is also implicated in autoantibody production in systemic lupus erythematosus [21].

We propose that vaccination with the BNT162b2 mRNA vaccine may have induced glomerulonephritis in our patients in two combined ways. This vaccine as well as the mRNA-1273 vaccine causes a strong tumor necrosis factor *α* response [13, 14]. This cytokine has been shown to aggravate glomerulonephritis in a mouse model of ANCA vasculitis and may have inflamed smoldering glomerular disease in our patients [22]. Additionally, the mRNA vaccine could have stimulated autoantibody production by activating ANCA-producing B cells, either via TLR7 or via cytokines produced by type 1 helper T cells [13, 14, 23]. Of note, de novo vasculitis has also been described after vaccination with the vector-based AZD1222 (Oxford-AstraZeneca) vaccine [9]. Finally, we cannot exclude that disease activation following vaccination happened by coincidence.

How to proceed with SARS-CoV-2 vaccination in a vasculitis patient treated with rituximab as maintenance therapy? We suggest that disease activity be monitored clinically and by means of ANCA titer immediately before scheduled vaccination. If there are indicators of risk of relapse, vaccination should be withheld and rituximab should be continued. B-cell recovery is a risk factor for relapse as well as a prerequisite for a good antibody response to immunisation. We also recommend either self-monitoring or, better, a clinical check-up one week after vaccination. In addition, it may be prudent for patients with ANCA-associated vasculitis to avoid mRNA vaccines because they could, in theory, aggravate glomerulonephritis and stimulate ANCA production. We think the vector vaccine Ad26.COV2.S (Johnson & Johnson) would be a good option for these patients because only one injection is necessary and immunosuppression can be reintroduced soon.

## Figures and Tables

**Table 1:** Clinical data and blood and urine test results of the two patients.

	Patient 1	Patient 2		Reference value
Age	54 years	78 years		
Sex	Female	Female		
Height	169 cm	160 cm		
Weight	70.9 kg	62 kg		

	After 2. vac.	Before 1. vac.	After 2. vac.	
BVAS	17	0	12	0–63 Points

*Blood tests*				
Leukocytes	10.6 G/L	6.7 G/L	6.4 G/L	3.7–10.0 G/L
Hemoglobin	9.2 g/dL	13.2 g/dL	9.3 g/dL	12.0–16.0 g/dL
CRP	11.79 mg/dL	0.10 mg/dL	0.43 mg/dL	<0.50 mg/dL
Urea	9.3 mmol/L	12.0 mmol/l	34.8 mmol/L	2.8–7.2 mmol/L
Creatinine	187 *μ*mol/L	142 *μ*mol/L	728 *μ*mol/L	44–80 *μ*mol/L
MPO-ANCA (Phadia)	106 IU/mL	99 IU/L	>134 IU/mL	<5 IU/mL
SARS-CoV-2 S antibody titer	>2572 BAU/mL		81.45 BAU/mL	<0.82 BAU/mL

*Urine tests*				
Hematuria	++++	++	+++	1-++++
Protein/creatinine ratio	0.44 g/g	0.15 g/g	3.56 g/g	<0.11 g/g

vac, vaccination; BVAS, Birmingham Vasculitis Activity Score; CRP, C-reactive protein; BAU/ml, binding antibody units/ml. The ELECSYS Anti-SARS-CoV-2 S (Spike/RBD-Protein) test from Roche Company was used to measure SARS-CoV-2 antibodies.

**Table 2:** Summary of reported cases of ANCA vasculitis after SARS-CoV-2 vaccination.

Reference	Age/sex	Vaccine (doses)	ANCA	Organ involvement	Treatment	Outcome
Anderegg et al. [6]	81 y/female	mRNA-1273 Moderna (2)	PR3	Kidney, lung	Steroids, CYC, TPE	Recovery
Sekar et al. [7]	52 y/male	mRNA-1273 Moderna (2)	PR3	Kidney	Steroids, rituximab, CYC, hemodialysis	Chronic hemodialysis
Shakoor et al. [8]	78 y/female	BNT162b2 mRNA Pfizer-BioNTech (2)	MPO	Kidney	Steroids, rituximab	Recovery
Villa et al. [9]	63 y/male	AZD1222 Oxford-AstraZeneca (1)	MPO	Kindey, lung	Steroids, CYC	Recovery
Patient 1	54 y/female	BNT162b2 mRNA Pfizer-BioNTech (2)	MPO	Kidney, lung	Steroids, rituximab	Recovery
Patient 2	78 y/female	BNT162b2 mRNA Pfizer-BioNTech (2)	MPO	Kidney	Steroids, rituximab, hemodialysis	Chronic hemodialysis

CYC, cyclophosphamide; TPE, therapeutic plasma exchange.

## Data Availability

No data were used to support this study.
